# Chromosome and Linear Plasmid Sequences of a 2015 Human Isolate of the Tick-Borne Relapsing Fever Spirochete, *Borrelia turicatae*

**DOI:** 10.1128/genomeA.00655-16

**Published:** 2016-07-14

**Authors:** Luke C. Kingry, Dhwani Batra, Adam Replogle, Christopher Sexton, Lori Rowe, Benjamin M. Stermole, Anna M. Christensen, Martin E. Schriefer

**Affiliations:** a Division of Vector-Borne Diseases, Bacterial Diseases Branch, Centers for Disease Control and Prevention, Fort Collins, Colorado, USA; b Division of Scientific Resources, Biotechnology Core Facility Branch, Centers for Disease Control and Prevention, Atlanta, Georgia, USA; c Eglin Hospital, Eglin Air Force Base, Florida, USA

## Abstract

The sequences of the complete linear chromosome and 7 linear plasmids of the relapsing fever spirochete *Borrelia turicatae* are presented in this report. The 925,547 bp of chromosome and 380,211 bp of plasmid sequence were predicted to contain a total of 1,131 open reading frames, with an average G+C content of 29.7%.

## GENOME ANNOUNCEMENT

Tick-borne relapsing fever is a zoonotic disease characterized by recurring febrile episodes associated with high levels of spirochetemia, followed by afebrile periods when little or no spirochetes can be detected in the blood (1, 2). Tick-borne relapsing fever is caused by relapsing fever group borreliae that includes *Borrelia hermsii*, *Borrelia parkeri*, and *Borrelia turicatae* in North America. *B. turicatae* is transmitted to humans by the soft-bodied *Ornithodoros turicata* tick vector (1, 2). *Borrelia* genomes are characterized by a long linear chromosome (~1 Mb) and several linear and circular plasmids (3). Relapsing fever group *Borrelia* plasmids encode particularly redundant sequences, making plasmid sequence assembly difficult; thus, little completed plasmid sequence is publicly available (4). Here, we provide the linear chromosome and 7 finished linear plasmid sequences from a culture of *B. turicatae* isolated from a patient in 2015.

A pure culture of *B. turicatae*, BTE5EL, was achieved in 2015 from the blood of an acutely ill and febrile patient. Total genomic DNA was isolated from mid-log-phase culture using the QIAamp DNA mini tissue protocol (Qiagen, Valencia, CA). DNA was sent to the Genome Sequencing Laboratory (CDC/Division of Scientific Resources, Atlanta, GA) and prepared for sequencing on the Pacific Biosciences RSII instrument (Pacific Biosciences, Menlo Park, CA). Using 1 single-molecule real-time (SMRT) cell (C4 chemistry; movie time, 240 min), a total of 966,463,251 bases were sequenced. The mean read length was 18,657 bp, with a mean read score of 0.86. *De novo* assembly was conducted using PacBio’s Hierarchical Genome Assembly Process (HGAP3, SMRTAnalysis 2.3.0) yielding 42 polished contigs with an average coverage of 270×. Manual inspection of the polished contigs revealed 8 finished linear contigs that ranged in length from 925,547 bp to 24,053 bp. The topology of the linear contigs was confirmed by sequence reads that read completely through the covalently closed telomeric ends at the 5′ and 3′ ends of each contig.

We present here a linear chromosome of 925,547 bp and 380,211 bp of finished plasmid sequence. The linear chromosome was predicted to contain 827 genes, 32 tRNA, and 3 rRNA loci. The average G+C content of the linear chromosome was 29.1%, and the average G+C content of the plasmids was 30.3%. Three hundred four genes were predicted to be carried on the plasmids, and no tRNA or rRNA loci were identified in the plasmid sequences.

These chromosome and plasmid data represent the second publicly available *B. turicatae* chromosome and only finished linear plasmid sequences for *B. turicatae*. The PacBio long-read platform is particularly suited for sequencing and assembling complex *Borrelia* genomes (5). It is anticipated that these data will be a useful tool for the assembly of incomplete plasmid data from existing and newly sequenced relapsing fever *Borrelia* spp. from shorter-read platforms. In addition, the availability of relapsing fever group *Borrelia* plasmid sequence will shed more light on the complex host-vector-pathogen interactions utilized by each of the relapsing fever group *Borrelia* species.

### Nucleotide sequence accession numbers.

The chromosome and plasmid sequences have been deposited in GenBank under accession numbers CP015629 to CP015636.
